# Sarcoid-like Granulomatosis Associated with Immune Checkpoint Inhibitors in Melanoma

**DOI:** 10.3390/cancers14122937

**Published:** 2022-06-14

**Authors:** Audrey Melin, Émilie Routier, Séverine Roy, Pauline Pradere, Jerome Le Pavec, Thibaut Pierre, Noémie Chanson, Jean-Yves Scoazec, Olivier Lambotte, Caroline Robert

**Affiliations:** 1 Department of Dermatology, Gustave Roussy, 114 rue Edouard-Vaillant, 94800 Villejuif, France; emilie.routier@gustaveroussy.fr (É.R.); severine.roy@gustaveroussy.fr (S.R.); 2 Service de Pneumologie et Transplantation Pulmonaire, Hôpital Marie-Lannelongue, Groupe Hospitalier Paris-Saint Joseph, 133 Av. de la Résistance, 92350 Le Plessis-Robinson, France; paulinepradere@gmail.com (P.P.); lepavec@gmail.com (J.L.P.); 3 Department of Medical Imaging, Gustave Roussy, 114 rue Edouard-Vaillant, 94800 Villejuif, France; thibaut.pierre@gustaveroussy.fr; 4 Department of Internal Medicine, Kremlin Bicêtre Hospital, 78 rue du Général Leclerc, 94270 Le Kremlin Bicêtre, France; noemie.chanson@aphp.fr (N.C.); olivier.lambotte@aphp.fr (O.L.); 5 Université Paris Saclay, AP-HP, 63 rue Gabriel Péri, 94270 Le Kremlin Bicêtre, France; jean-yves.scoazec@gustaveroussy.fr; 6 Department of Pathology, Gustave Roussy, 114 rue Edouard-Vaillant, 94800 Villejuif, France

**Keywords:** melanoma, immunotherapy, immune checkpoint inhibitor, granulomatosis, sarcoidosis, mTOR

## Abstract

**Simple Summary:**

Granulomatosis is commonly observed in patients receiving immune checkpoint inhibitors (ICIs) for melanoma and other cancers. Recognizing this reaction early is critical to avoid confusing it with cancer progression. Our objectives were to characterize instances of granulomatosis triggered by immunotherapy, to question their relationship with sarcoidosis, to explore their potential pathophysiological mechanisms and to address the possibility of an association between such events and response to immunotherapy. Our work is based on a retrospective study of 18 cases and on a thorough review of the literature of melanoma patients developing ICI-associated granulomatosis. After analyzing the clinical, histopathological and biological results, we propose that immuno-induced granulomatosis corresponds to an experimental sarcoidosis and should be considered as an ICI-associated effect rather than a severe immune-related event. Indeed, ICI-associated granulomatosis seems to be associated with a good ICI response and usually does not require ICI interruption or symptomatic treatment.

**Abstract:**

We aimed to review the clinical and biological presentation of granulomatosis associated with immune-checkpoint inhibitors (ICI) in patients with melanoma and to explore its association with classical sarcoidosis as well as with cancer response to ICI. To this end, a retrospective study on 18 melanoma patients with histologically proven ICI-induced granulomatosis over a 12-year period in a single center, as well as on 67 similar cases reported in the literature, was conducted. Results indicate ICI-induced granulomatosis is an early side effect (median time to onset: 2 months). Its clinical presentation, with predominant (90%) thoracic involvement, histopathological appearance and supposed underlying biology (involving the mTOR pathway in immune cells, Th17 polarization and TReg dysfunction) are indistinguishable from those of sarcoidosis. Moreover, it appears to be associated with ICI benefit (>65% objective response rate). Evolution is generally favorable, and symptomatic steroid treatment and/or ICI discontinuation are rarely necessary. ICI-associated granulomatosis is critical to explore for several reasons. Practically, it is essential to differentiate it from cancer progression. Secondly, this “experimental” sarcoidosis brings new elements that may help to address sarcoidosis origin and pathophysiology. Its association with ICI efficacy must be confirmed on a larger scale but could have significant impacts on patient management and biomarker definition.

## 1. Introduction

Immunotherapy using immune checkpoint inhibitors (ICIs) has been a major therapeutic breakthrough in the treatment of melanoma over the past decade, and its indications are now being gradually broadened to a wide spectrum of other cancers. The anti-CTLA-4 ipilimumab was the first ICI approved for metastatic melanoma, with an objective response rate of 11–15% and a median overall survival (OS) of 20 months, whereas anti-PD-1s, such as pembrolizumab or nivolumab, demonstrated an objective response rate around 43% and a median OS of 37 months [1,2]. Finally, combined anti-CTLA-4 and anti-PD-1 (ipilimumab and nivolumab) has a response rate around 58% and a median OS of 72 months [3]. Nevertheless, the enthusiasm generated by these latter results must be balanced by the 60% risk of grade 3/4 toxicity associated with the immunotherapy combination [4].

Among ICI adverse events, immunological reactions referred to as “sarcoidosis” or granulomatosis are increasingly reported. These ICI-induced granulomatoses have been described with anti-PD-1, anti-CTLA-4 as well as with their combination. In fact, a non-random association between sarcoidosis and cancer, and melanoma in particular, has been reported for a long time [5]. First, sarcoidosis is associated with an increased risk of developing cancer, particularly melanoma [6,7]. The underlying pathophysiological mechanisms proposed for this association remain unclear. On the other hand, granulomatosis and sarcoidosis seem to be more frequent in patients with cancers, including melanoma [8,9]. These melanoma-associated granulomatous reactions were reported before the advent of ICI and were described as a particular T-cell-mediated immune response occurring in response to tumor cells, either at their contact, in the tumor-draining lymph nodes or at distant sites [10,11,12]. In some cancer types, such as lymphomas and gastric cancers, sarcoidosis is considered a favorable prognostic factor [13]. Finally, granulomatous reactions have been reported during various therapies, especially when using immunomodulatory drugs such as antiretroviral therapies, TNF-alpha antagonists, interferon therapies, immunotherapies and targeted therapies. More rarely, it has also been reported with anti-CD20, anti-CD25, anti-IL6 and anti-IL12/23 antibodies. In melanoma patients, aside from the cases associated with IFN-alpha and more recently with ICI, drug-induced granulomatous reactions have also been reported with chemotherapy as well as with targeted anti-BRAF and anti-MEK therapies [14]. 

The diagnosis of sarcoidosis is based on a combination of clinical, biological and radiological findings, the demonstration of non-caseous granuloma on histology and the exclusion of other causes of granulomatosis, including iatrogenic causes. Therefore, since ICI-induced granulomatosis is by definition secondary to a therapy, it should, in theory, be considered as an iatrogenic event and should therefore represent an exclusion criteria for the diagnosis of sarcoidosis. 

Currently, the proposed mechanisms underlying the occurrence of sarcoidosis involve both innate and adaptive immune responses to a yet unknown antigen resulting in the accumulation of activated CD4 T cells and M1 macrophages in the affected organs with the formation of the characteristic epithelioid granuloma. 

In addition, several genetic and environmental factors have been linked to sarcoidosis. 

Indeed, some familial forms of sarcoidosis have been reported, and various genes are associated with the phenotypes, severity and clinical course of sarcoidosis. In particular, there is an association between clinical presentation of sarcoidosis and several genes coding for MHC (HLA A, B, DQ and DR) or for proteins involved in apoptotic, enzymatic, regulatory or immune functions (NOD2, NOTCH4, BTNL2, TAP2, TNF, VEGF, PTGS/COX2, IL1A, IL23R, CCR5, etc.) [15,16,17]. 

In addition, two types of environmental factors have been identified, one microbial, the other work-related. Thus, many sources support the involvement of mycobacteria and/or propionibacteria (*Proprionibacterium acnes* or *granulosum*) in the formation of sarcoidosis. Some studies reveal a marked overlap between the transcriptomic signatures of tuberculosis and sarcoidosis [18]. Others have reported positive mycobacterial PCR in 26% of patients with sarcoidosis and suggest that residual mycobacterial catalase-peroxidase could be the tissue antigen triggering sarcoidosis [19,20]. However, because mycobacteria cultures remain negative, an active infection is excluded [21]. Other studies found a positive PCR for *P. acnes* and *P. granulosum* in the lymph nodes of patients with sarcoidosis, and a specific response directed against a *P. acnes* antigen was reported in Japanese sarcoidosic subjects [22,23]. Moreover, sarcoidosis-like granulomatosis could be induced through increased IL17 secretion in *Propionibacterium acnes* carrier mice models [24]. Nevertheless, the pathogenic role of *P. acnes* in sarcoidosis remains unclear, as other studies suggest that *P. acnes* is a commensal organism frequently detected in the lungs and lymph nodes of healthy individuals [25]. Thus, sarcoidosis does not appear to be caused by an active infection, and the exact microbial pathogenic mechanisms remain unknown.

Finally, certain environmental occupations or exposure to toxic substances have been associated with sarcoidosis, such as agricultural employment, insecticides or microbial (mold/mildew) bioaerosols [26,27].

Therefore, to date, the pathophysiology of sarcoidosis remains unclear. The main hypothesis is that sarcoidosis represents an immune reaction to various environmental factors that trigger inflammation in patients with a predisposed genetic background. 

In this study, we analyzed a series of patients who developed granulomatosis during anti-CTLA-4 and/or anti-PD-1 immunotherapy administered for melanoma and performed a review of the literature on this subject. 

The aim of this study was to characterize immuno-induced granulomatosis and to evaluate the impact of this event on the outcome of melanomas. Our secondary objective was to propose pathophysiological hypotheses that could explain the occurrence of ICI-induced granulomatosis in order to see whether these reactions could be defined as sarcoidoses. 

## 2. Materials and Methods

We first conducted a single-center retrospective observational study (Gustave Roussy Cancer Campus) from May 2007 to October 2019 to include all adult patients who developed a granulomatosis reaction during ICI treatment for melanoma. Patients were eligible for inclusion if they had a confirmed diagnosis of melanoma, received anti-PD-1 and/or anti-CTLA-4 immunotherapy and developed a granulomatosis during treatment. Our diagnostic criteria for sarcoidosis-like granulomatosis were: suggestive radiological and/or clinical signs confirmed by the presence of tuberculoid granulomas without caseous necrosis on pathological examination and the absence of other causes of granulomatosis, such as infectious disease. The diagnosis of sarcoidosis was systematically validated in a multidisciplinary board meeting involving internists, anatomopathologists, oncologists, radiologists and pneumologists.

Exclusion criteria included patients presenting with granulomatosis prior to the start of immunotherapy as well as lack of histological confirmation of granulomatosis. 

In order to compile the data, we interrogated the Gustave Roussy computer software “AMBRE”, which includes all the patients treated in the institution, and more particularly the search module entitled “AMBRE SEARCH” using the search criteria: “melanoma”, “anti-CTLA-4”, “anti-PD-1”, “granulomatosis”, “sarcoidosis”. 

Tumor responses to immunotherapy were evaluated by computed tomography (CT) or positron emission tomography–computed tomography (PET/CT) according to the Response Evaluation Criteria in Solid Tumours (iRECIST) and Positron Emission Tomography Response Criteria in Solid Tumors (PERCIST) evaluation criteria, respectively.

A centralized review of all CT and FDG PET/CT imaging was performed at the time of collection, both in terms of imaging description of granulomatosis and oncologic responses.

Continuous data are presented using median, minimum and maximum values. Categorial data are described using percentages. Overall survival, defined as the time from diagnosis of granulomatosis to death from any cause, was estimated using log-rank survival analysis. 

Following that, we reviewed the published literature on immunotherapy-treated melanoma patients who developed ICI-induced granulomatosis and whose oncological outcome was reported. The PubMed search equation performed on 26 February 2022 was as follows: (“sarcoidosis” OR “Sarcoid-like” OR “granulomatosis”) AND (“ipilimumab” OR “nivolumab” OR “pembrolizumab” OR “Anti-CTLA-4” OR “anti-PD-1” OR “checkpoint inhibitors” or “immune checkpoint” OR “melanoma treatment”).

## 3. Results

**Figure 1:**
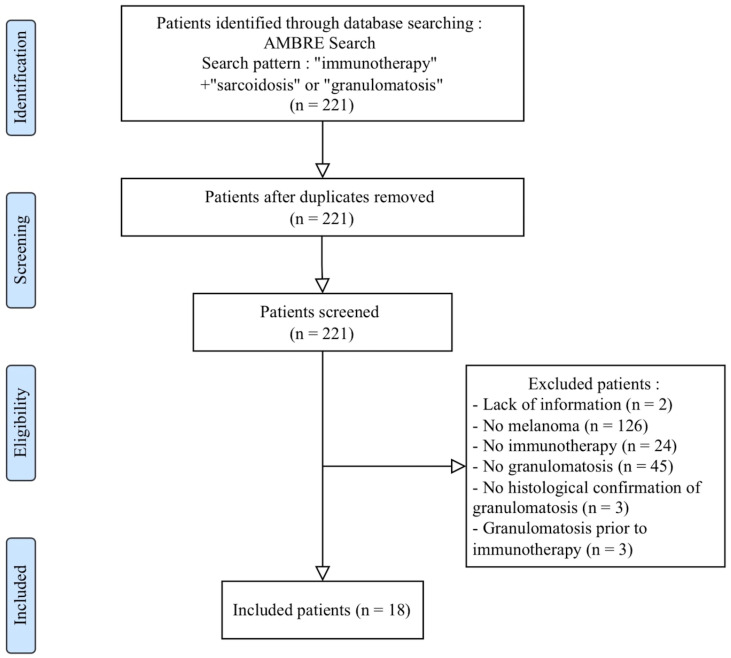
PRISMA study–Patient selection flow chart.

Eighteen patients were included with a mean age at diagnosis of melanoma of 47 years [22–73 years] (Figure 1). 

The female-to-male ratio was 1.6. Patient characteristics are reported in Table 1. Three patients (16.7%) had stage III and 15 patients (83.3%) stage IV melanoma. Sixteen patients received immunotherapy for unresectable melanoma: twelve as a 1st-line treatment and four as 2nd- or 3rd-line treatment. Two patients with stage IIIC and stage IV melanoma (TxN0M1d) received adjuvant immunotherapy after lymph node resection and neurosurgery followed by conformal radiotherapy under stereotactic conditions, respectively. Two patients received intravenous ipilimumab, seven received intravenous anti-PD-1 alone, and nine patients underwent dual immunotherapy with anti-PD-1 nivolumab + anti-CTLA-4 ipilimumab.

All patients developed clinical (55.6%), biological (55.6%) or radiological (94.4%) signs consistent with sarcoidosis shortly after the introduction of immunotherapy. 

The clinical manifestations, observed in 10 patients, appeared concomitantly with the radiological signs within a median period of 2 months (1–11) following the introduction of immunotherapy.

Six patients developed respiratory symptoms such as dyspnea, cough or chest pain. In addition, five out of seven patients had lymphocytic alveolitis on bronchoalveolar lavage fluid (BAL) analysis, and four out of thirteen patients showed abnormalities on pulmonary function tests, including obstructive syndrome in one, restrictive syndrome in one and diffusion disorder in two patients. Chest CT scans were compatible with sarcoidosis and revealed mediastinal adenopathies in all patients and interstitial involvement in nine patients. However, no pulmonary fibrosis was observed. In addition, hepatic (grade 2–3 cytolysis), ophthalmologic (uveitis and keratoconjunctivitis) and dermatologic (sarcoidosis papules and nodules) involvement were present in 11.1% of cases. Finally, one patient (5.6%) presented an isolated grade 2 acute renal failure related to granulomatosis. 

Laboratory tests showed lymphopenia in 5/18 patients, anicteric cholestasis in 4/18, hypergammaglobulinemia in 1/7 and elevated angiotensin-converting enzyme in 2/8. No hypercalcemia was reported, and the QuantiFERON was systematically negative.

Granulomatosis was suspected on imaging in 17 patients, during a routine oncological re-evaluation in 8 patients and when exploring biological or clinical abnormalities, eventually linked to granulomatosis, in 9 patients. Positron emission tomography (PET)–computed tomography (CT), performed in 14 patients, systematically revealed inflammatory mediastino-hilar or internal mammary nodes, pulmonary infiltrate, nodules of the upper lobes or pleural lesions. Subcutaneous lesions of various topographies were also found in 4 patients. CT scanners showed mediastinal lymph nodes, scissural nodules and subcutaneous nodules in 73% of cases (11 of 15 patients). For three patients, progression or pseudo-progression were initially suspected. Overall, the onset of radiological signs of granulomatosis was predominantly precocious, with a median time of 2 months (1–10) following the start of immunotherapy.

Histological confirmation of granuloma was obtained by biopsy of mediastinal nodes in 13 patients, liver in 2, kidney in 1, skin in 1, and supraclavicular nodes in 1. The median time from initiation of treatment to histological diagnosis was 4 months (1–11 months).

Ten patients had their management modified. Seven patients (39%) received systemic corticosteroid therapy with a median dose of prednisone of 1 mg/kg/day, six of whom had symptomatic granulomatosis and one of whom had a concomitant immuno-induced neuropathy. The decision to discontinue or maintain immunotherapy was made after a case-by-case discussion, weighting, in a multidisciplinary board meeting, the risk versus the benefit of continuing ICI treatment. It depended on the severity of the granulomatosis and expected oncologic benefit of pursuing ICI treatment. Immunotherapy was discontinued in seven patients after a decision by the immunotoxicity review committee. Of these, five patients were receiving dual immunotherapy and two were able to continue the anti-PD-1 therapy alone. In total, four of the above ten patients simultaneously received corticosteroids and stopped ICI treatment. All granulomatosis-related organ abnormalities, clinical or biological, eventually regressed within a median of 17 weeks (2–43.3).

Regarding oncologic response at the time of the diagnosis of granulomatosis, 12 of the 16 patients (75%) with unresectable or metastatic melanoma had an objective response: 5 complete responses, 2 complete or partial responses depending on the method of assessment and 5 partial responses. Two patients had stable disease, and only two patients had a progressive disease. Overall, 87.5% (14/16) of the patients had disease control. When we analyze the response by treatment type, both patients treated with ipilimumab showed oncologic stability, 89% (8/9) of the patients treated with the ipilimumab + nivolumab combination presented with an objective response (3 complete responses, 4 partial responses and 1 partial or complete response based on reassessment modality) and 1 patient progressed. With anti-PD-1 monotherapy, 4 patients (80%) showed an objective response (2 complete responses, 1 partial response and 1 partial or complete response according to the method of reassessment), and 1 patient experienced progression. Furthermore, all patients treated in the adjuvant setting were free of relapse. Changes in LDH levels were noted during granulomatosis. Among 11 patients with metastatic or unresectable melanoma who had blood LDH measured, 5 had an elevated LDH level prior to ICI. All of them normalized this dosage during the treatment and the sarcoidosis reaction while they developed a complete or partial response. Two patients had elevated LDH levels at the time of granulomatosis. For one patient, it likely resulted from hepatic cytolysis secondary to granulomatosis, and from melanoma progression for the other. 

**Figure 2:**
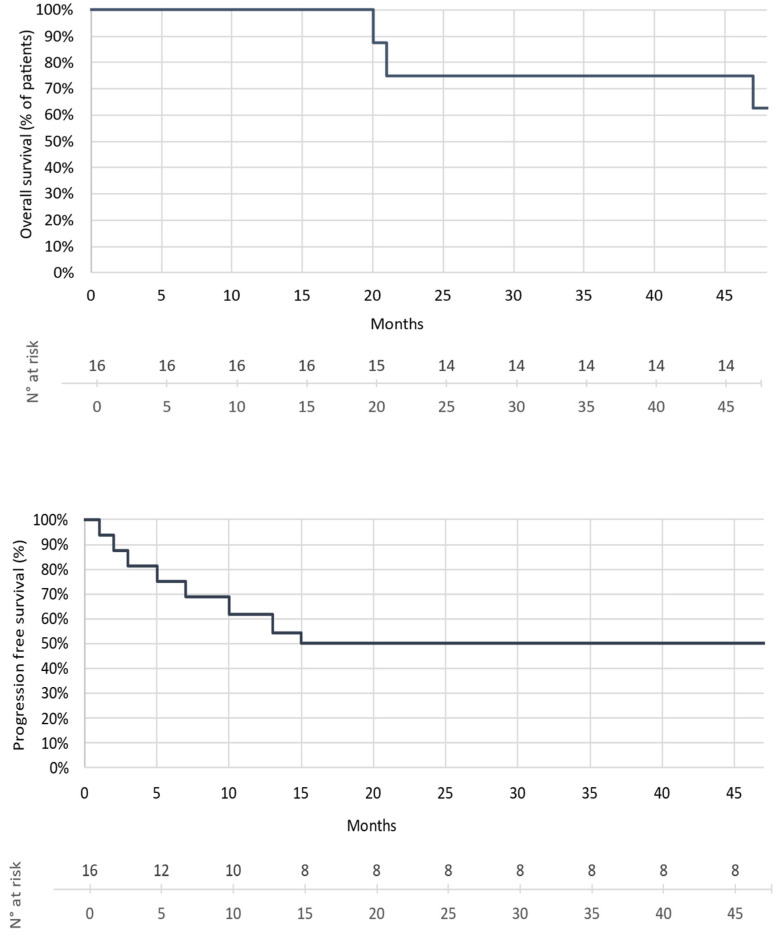
Kaplan Meier: OS and PFS estimates (median follow-up: 22 months).

Regarding the patients’ outcome at the time of data collection, i.e., within a median follow-up time of 22 months (6–50 months), eight had progressed, and of these, three had died of melanoma. In addition, all patients treated in the adjuvant setting were free of relapse. The overall survival rate of the patients with metastatic/unresectable melanoma was 75%, and their progression-free survival rate was 50% (Figure 2).

In the second part of our study, we conducted a literature review of cases of immuno-induced granulomatosis during melanoma management. A total of 183 articles matched the search equation. Of these, 45 were related to cases of granulomatosis occurring during ICI treatment for melanoma with information on oncologic follow-up [28,29,30,31,32,33,34,35,36,37,38,39,40,41,42,43,44,45,46,47,48,49,50,51,52,53,54,55,56,57,58,59,60,61,62,63,64,65,66,67,68,69,70,71,72]. A total of 67 patients were described, with a majority of men (55%) and a mean age at diagnosis of melanoma of 58 years. Forty-two patients were receiving ICIs for unresectable melanoma, and 25 were receiving them as adjuvant therapy. Patient characteristics are reported in Table 2. The median time to onset of granulomatosis-related symptoms following the introduction of immunotherapy was 3 months (1–43). Thoracic involvement was present in 91% of cases, followed by dermatological and lymph node involvement in 48% and 12%, respectively. Other organs were less frequently affected. Twenty-five patients (37%) received systemic corticosteroid therapy, and immunotherapy was discontinued in thirty-three (49%) patients. Radiological reassessment of the granulomatous reaction was provided for 62 (93%) patients. Of these, 87% (54/62) showed partial or complete regression of the granulomatosis radiological signs, 13% (8/62) were stable, and no further radiological deterioration was recorded. Regarding the oncologic response at the time of granulomatosis, 29 patients (69%) out of 42 followed for unresectable melanoma had an objective response, and 34 (81%) had disease control. Eight (19%) showed progression. In addition, 3 out of the 25 patients treated in adjuvant therapy had relapsed. Regarding the oncological response at the time of the last re-evaluation, i.e., after a mean duration of 8 months, 24 (57%) of the 42 patients followed up for unresectable melanoma had an objective response, and 28 (67%) had disease control. Fourteen patients (33%) had progressed. Three out of 25 patients treated in adjuvant therapy had relapsed. 

## 4. Discussion

**Figure 3:**
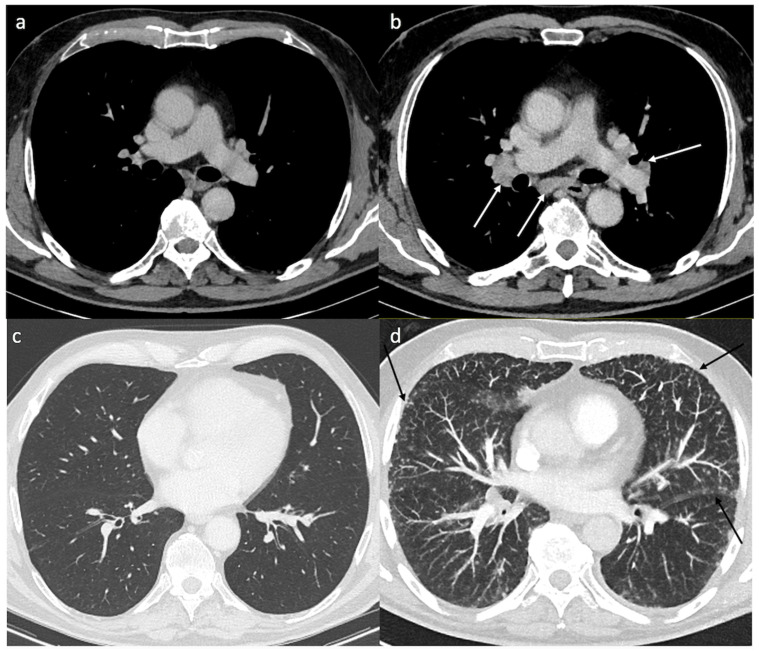
Typical findings of pulmonary sarcoidosis. Axial contrast enhanced computed tomography (CT) shows no mediastinal lymph node enlargement (**a**) and no parenchymal disease (**c**) before treatment. Five months after immunotherapy, axial enhanced CT shows typical bilateral and symmetric hilar and subcarinal lymph nodes (**b**) and multiple micronodules with a perilymphatic distribution (subpleural and perifissural nodules) (black arrows-**d**).

Our series and the literature review present similar characteristics, namely the appearance, early after ICI initiation, of clinical, biological and radiological symptoms associated with a granulomatous reaction and preeminent thoracic involvement (Figure 3). These results also suggest that the appearance of granulomatosis during ICI treatment could be associated with a clinical benefit. Indeed, the response rates observed in our series as well as in the literature (75% and 69%, respectively) are higher than the ones usually reported with ICI (around 43% for anti-PD-1 and 58% with the combination of ipilimumab + nivolumab). In addition, it is noteworthy that a favorable outcome was observed in our cohort of patients in spite of poor prognostic factors such as multiple metastatic locations and high LDH levels. 

The majority of the cases of granulomatosis were not severe and did not necessitate ICI treatment interruption. The management of ICI-associated granulomatosis is usually discussed on a case-by-case basis. As more and more cases are reported, and patient outcomes are rather reassuring, physicians are less prone to stop ICI, and it seems reasonable to advise corticosteroid use only for symptomatic cases [14].

As the use of immunotherapies is rapidly increasing, it is essential for clinicians to diagnose this lateral effect of immunotherapy in order to avoid diagnostic errors. Indeed, one of the main issues when ICI-associated granulomatosis occurs is the differential diagnosis with progression or pseudo-progression of the melanoma. 

The actual frequency of granulomatous reactions following melanoma immunotherapy remains uncertain, ranging from 2% to 22.2% of cases, according to several studies [47,73,74]. In our series and our review of the literature, it seems that mediastinal lymph node involvement is particularly frequent. Harsha Tirumani et al. described mediastinal and hilar nodes in 5% of patients treated with anti-CTLA-4 that were asymptomatic and spontaneously resolutive [75]. However, appearance or enlargement of mediastinal nodes can of course be due to a progressive melanoma or to a pseudo-progressive disease that will eventually respond to ICI [76]. Although there is ongoing research in nuclear medicine to target specific immune cells in order to discriminate between an inflammatory response such as pseudo-progression and a true progression, to date, there is no marker to distinguish these two entities [77]. Therefore, although radiological interpretation may suggest a diagnosis of granulomatosis, these different entities remain challenging to distinguish. Multidisciplinary evaluation combined with pathological confirmation is of critical importance before considering a patient to be progressing, as it can have deleterious consequences in term of melanoma management. Indeed, wrongly considering that a patient is progressing when he has granulomatosis would lead to a therapeutic error by prescribing another anti-cancer treatment when the patient may be responding to immunotherapy. In terms of management, if the mediastinal granulomatous lymph nodes are isolated and asymptomatic, there is no need to interrupt ICI treatment or to prescribe systemic steroids. If there are signs of interstitial pneumonitis and/or respiratory symptoms in addition to the lymph node involvement, a functional respiratory test should be performed, and bronchoalveolar lavage should be considered after discussion with pneumologists or internists. Depending on these results, interruption of ICIs and systemic steroids can be advised. In all cases, a close follow-up with clinical evaluation, functional tests and a CT scan should be performed. In many centers, multidisciplinary board meetings dedicated to immune toxicity have been created and can help in the difficult situations. In the case of discontinuation of ICIs, it is advised to ensure that there is a real oncological progression before considering a re-challenge with ICIs or a new treatment initiation.

While anti-PD-1 +/− CTLA-4 immuno-induced granulomatous lesions have often been described as authentic sarcoidosis because of their clinical, biological, histological and radiological presentation and their sensitivity to corticosteroid therapy, we can challenge this assertion and refer to the definition of sarcoidosis. It relies on three criteria: (1) a compatible clinical and radiologic presentation, (2) pathologic evidence of noncaseating granulomas and (3) exclusion of other diseases with similar findings, such as infections or malignancy [78,79]. 

Indeed, the diagnosis of sarcoidosis is based on the exclusion of other possible causes of granulomatosis, notably cancers or iatrogenic causes. Thus, granulomatous reactions occurring under immunotherapy for melanoma should not theoretically be qualified as true sarcoidosis. However, we propose to address this question after exploring the current knowledge on sarcoidosis pathophysiology as well as on granulomatosis induced by anti-tumor immunotherapy. 

The pathophysiology of sarcoidosis is complex and still inadequately comprehended. Several immunological hypotheses based on different mathematical or experimental models have been formulated in the past few years without being able to establish, with certainty, the exact role and order of the immunological events [80]. As in many inflammatory diseases, this pathology is characterized by a close collaboration between innate and adaptive immunity following environmental antigenic exposure in a favorable genetic context [81,82]. After antigenic stimulation, whose infectious origin (e.g., *mycobacteria* or *propionibacteria)* is uncertain, the antigen-presenting cells (macrophages, dendritic cells) induce a Th1/Th17 oriented immune T-cell response as well as a dysfunction of T Reg lymphocytes, which is triggered by numerous cytokines and proinflammatory chemokines [83,84,85].

Histology reveals a non-caseating epithelioid granuloma associated with CD4+ T cell infiltration in the affected organs. In peripheral blood, there is a consistent depletion of CD4+ T cells and B cells. CD4+ T cells in blood and BAL are predominantly polarized Th1 (express IFN-γ, TNF-α and IL-2), to a lesser extent Th17 and Th17.1 (synthesize IL17, IFN-γ, CCR6) [86]. The Th17/T Reg ratio is increased in the peripheral blood and BAL of patients with sarcoidosis. This disbalance between these populations is further marked by a decrease in the suppressive function of T Reg lymphocytes [87,88]. Moller et al. point out the numerous gaps concerning the pathophysiological aspects of sarcoidosis, in particular the role of macrophages and their polarization, as well as the role of T Reg, Th17 and B lymphocytes, and attribute this lack of knowledge to the inability to create an animal or in vitro model on account of insufficient data on the concerned antigens [89].

The specific immunological pathways responsible for granuloma formation in cancer have not been established. One hypothesis is that the immune response occurring during cancer immunosurveillance induces the activation of pro-inflammatory innate immune cells as well as Th1 and Th17 effector T cells, which are involved in granulomatous reactions. However, the possible causative tumor-associated antigens remain unknown. Concerning the potential role of ICIs, several hypotheses can be formulated [90]. First, anti-CTLA-4 immunotherapy restores the activation, proliferation and polarization of CD4+ T cells into pro-inflammatory lymphocytes such as Th1 and Th17 and is responsible for an alteration of T Reg cell function [91]. It also leads to an increase in Th17 cells in the peripheral blood of patients with metastatic melanoma [92]. Moreover, T Regs and Th17 cells found in lymph nodes of patients with sarcoidosis have decreased CTLA-4 expression, leading to an altered balance of T Helper/T Reg and to an increased proportion of Th17. This observation suggests a critical role of CTLA-4 in sarcoidosis pathogeny and is in agreement with the finding that anti-CTLA-4 treatment can increase Th17 and induce sarcoidosis [93]. 

For anti-PD-1-induced granulomatosis, the involvement of the mTOR signaling pathways in lymphocytes and macrophages is suspected [94]. The pathophysiological link between the PD-1/PD-L1 pathway and both T Reg and Th17 lymphocytes has been established, notably in the field of gynecology-obstetrics through studies on maternal–fetal tolerance and in particular in the article by Zhang et al. on treating pre-eclampsia [95,96]. This study highlights the essential role of PD-1/PD-L1 in the regulation of T Reg and Th17 cells. Indeed, the PD-1/PD-L1 signaling pathway is associated with an increase in T Reg lymphocytes, whereas its blockage leads to an immunological response similar to that found in sarcoidosis. In fact, the PD-1/PD-L1 interaction causes a decrease of PI3K expression in T lymphocytes, along with a decrease of AKT and mTOR, leading to a T Reg-type polarization of the lymphocytes. Conversely, in the case of PD-1/PD-L1 dysfunction, increased expression of PI3K/AKT/mTOR induces a conversion of T Reg cells into Th17 cells. Therefore, when the PD-1/PD-L1 pathway is blocked (i.e., during anti-PD-1 immunotherapy), the mTOR pathway is activated, Th17 lymphocytes are abundant, and T Reg lymphocytes are less present. Moreover, Th17 (CCR6 + CCR4 + CXCR3 secreting IL17) and Th17.1 (CCR6 + CCR4-CXCR3 + CCR10 secreting IFN gamma and IL17) lymphocytes are involved in the development of sarcoid-type granulomatous reactions, and the latter cell population is notably found in BAL. Both populations are also found in patients with melanoma and ICI-induced granulomatosis [86,97].

Thus, blocking the PD-1/PD-L1 pathway induces, via activation of the mTOR pathway in lymphocytes, dysfunction of T Reg and a Th17 type T-cell response, leading to granuloma formation through IL17 secretion [98,99]. This hypothesis is supported by the work of Linke et al. concerning the role of the mTOR pathway in macrophages, whose activation is associated with active sarcoidosis [100]. It reports the development of granuloma in a mouse model with constitutive activation of mTORC1 in myeloid cells and thus in macrophages (obtained by deletion of tsc2). In vivo, mTORC1 activation induces excessive granuloma formation, and its inhibition, by adding everolimus, leads to clinical resolution of the granulomas. Furthermore, this study indicates, via gene set enrichment analysis (GSEA), that both activation of mTORC1 and macrophage proliferation are associated with disease progression in humans with active sarcoidosis. Overall, blockading of the PD-1/PD-L1 pathway by immunotherapy may promote granuloma formation through activation of the PI3K/mTOR pathway in macrophages and T cells, resulting in regulatory T-Reg-cell dysfunction and polarization of lymphocytes into IL17-secreting Th17, similarly to what can be described in sarcoidosis. 

This suggests that prospective studies using mTOR inhibitors could be evaluated in symptomatic immune granulomatosis, especially since mTOR inhibitors also have anti-cancer properties.

Altogether, granulomatosis induced by ICIs and real sarcoidosis not only share clinical and pathology criteria, but they also have common biological mechanisms involving Th1 and Th17 and possibly the PI3K/mTOR pathway in macrophages and T cells. Nevertheless, the nature of the triggering antigens remains mysterious in both sarcoidosis and ICI-induced granulomatosis. Finally, these hypotheses need to be further explored and demonstrated by more in vitro and in vivo studies.

As a result, we propose that ICI-associated granulomatosis could be considered as real experimental sarcoidosis that is facilitated or amplified by the use of ICI. The latter could, in a way, lower the threshold for the appearance of sarcoidosis in certain individuals.

Finally, the immunological events detailed above leading to granuloma formation and immuno-induced granulomatosis are also associated with anti-tumor activity, which could explain the positive anti-tumor impact observed in cases of immuno-induced granulomatosis. Thus, the cascade of immune events induced by ICIs and leading to sarcoidosis in a subpopulation of patients could in fact represent a virtuous anti-tumor loop.

## 5. Conclusions

ICI-induced granulomatosis is an insufficiently documented side effect occurring in approximately 5% of patients, with a clinical, biological, radiological and histological presentation comparable to sarcoidosis. ICI-induced granulomatosis can affect multiple organs, although bronchopulmonary involvement clearly dominates. Patients may be asymptomatic or have clinical or laboratory abnormalities related to the affected organs. However, because imaging interpretations (radiography, CT, or PET/CT) are generally unable to distinguish malignant from inflammatory processes, misdiagnoses of recurrent, progressive or pseudo-progressive disease are likely to be misleading. Therefore, multidisciplinary discussion and histologic confirmation of each case are required, and additional nuclear medicine studies to differentiate progression, intra-tumor lymphocytic infiltration or granulomatous reaction should be considered. Management of this adverse event should be discussed on a case-by-case basis to assess the benefit of systemic corticosteroid therapy and to consider discontinuation of immunotherapy. This decision is all the more complex while the spontaneous evolution (remission, stabilization or evolution towards fibrosis) of this immuno-induced reaction remains unknown. Thus, the use of corticosteroids should be reserved for symptomatic cases only, in order to avoid excessive administration. Similarly, discontinuation of immunotherapy must be non-systematic and justified through dedicated multidisciplinary meetings on the management of immuno-induced toxicities. In case of recommended discontinuation, ensuring real oncological progression is encouraged before considering further alternative treatments.

Our review of the literature shows that sarcoidosis and immunotherapy-induced granulomatosis share a common pathophysiology, though it is still insufficiently comprehended. This adverse event would appear to be an immune response involving the mTOR pathway in macrophages and CD4 T cells leading to a polarization into Th1 and Th17 and to a dysfunction of the T Reg cells. Genetic polymorphisms and microbiological factors (e.g., *mycobacteria*, *propionibacteria*) associated with sarcoidosis have, to our knowledge, never been studied in cases of immuno-induced granulomatosis, in which the facilitating factors remain unknown. 

Finally, these results suggest that the development of granulomatosis in patients treated with immunotherapy for melanoma is associated with clinical benefit. The occurrence of granulomatosis may thus be an early marker of tumor response, but larger prospective studies are needed to validate this hypothesis. 

## Figures and Tables

**Table 1:** Gustave Roussy’s patient characteristics.

Patient Characteristics (N = 18)	
**Gender**	N = 18
Female	11 (61%)
**Age**	
Mean age at melanoma diagnosis (min–max) (years)	47 (22–73)
**Melanoma medical history**	
Melanoma type	
Cutaneous	14 (78%)
Mucosal	2 (11%)
Unknown primitive	2 (11%)
Oncological approach	
Adjuvant	2 (11%)
Metastatic	16 (89%)
1st line of treatment	12 (75%)
2nd or 3rd line of treatment	4 (25%)
Type of immunotherapy	
Anti-CTLA-4 monotherapy	2 (11%)
Anti-PD-1 monotherapy	7 (39%)
Anti-CTLA-4 + Anti-PD-1 combined	9 (50%)
**Immune-induced granulomatosis**	
Clinical features	10 (56%)
Median time since ICI initiation (min–max) (months)	2 (1–11)
Thoracic	6 (33%)
Dermatological	2 (11%)
Ophthalmologic	2 (11%)
Hepatic	2 (11%)
Renal	1 (6%)
Radiological features	
Consistent radiological signs	17 (94%)
Median time since ICI initiation (min–max) (months)	2 (1–10)
18-FDG PET/CT; *n* = 14	
Mediastino-hilar nodes	14 (100%)
Interstitial involvement	6 (43%)
Subcutaneous nodules	4 (29%)
CT scanner; *n* = 15	
Mediastino-hilar nodes	11 (73%)
Interstitial involvement	1 (7%)
Subcutaneous nodules	1 (7%)
Biological features	
Lymphopenia	5 (28%)
Anicteric cholestasis	4 (22%)
Hypergammaglobulinemia; *n* = 7	1 (14%)
Elevated angiotensin converting enzyme; *n* = 8	2 (25%)
Hypercalcemia	0 (0%)
Histological confirmation	18 (100%)
Median time since ICI initiation (min–max) (months)	4 (1–11)
Therapeutic management	
Systemic corticosteroids	7 (39%)
Discontinuation of immunotherapy	7 (39%)
Granulomatosis outcome	
Regression of clinical/biological features	18 (100%)
Radiological outcome of granulomatosis; *n* = 16	
Stability	8 (50%)
Partial or complete regression	8 (50%)
**Oncological outcome at the time of granulomatosis**	
Patient in adjuvant condition	N = 2
Relapse	0 (0%)
Patients in metastatic stage	N = 16
Objective response	12 (75%)
Complete response	5 (42%)
Complete or partial response	2 (17%)
Partial response	5 (42%)
Stability	2 (13%)
Progression	2 (13%)
**Oncological outcome at data collection**	
Mean follow-up time for melanoma (min–max) (months)	22 (6–50)
Patient in adjuvant condition	N = 2
Relapse	0 (0%)
Patients in metastatic stage	N = 16
Objective response	8 (50%)
Complete response	5 (63%)
Partial response	3 (38%)
Stability	0 (0%)
Progression	8 (50%)
Death	3 (38%)

**Table 2:** Literature Review–Patient characteristics.

LITERATURE REVIEW: PATIENT CHARACTERISTICS (N = 67)	
**Gender**	N = 67
Male	37 (55%)
**Age**	
Mean age at melanoma diagnosis (min–max) (years)	58 (26–83)
**Melanoma medical history**	
Oncological approach	
Adjuvant	25 (37%)
Metastatic	42 (63%)
Type of immunotherapy	
Anti-CTLA-4 monotherapy	19 (28%)
Anti-PD-1 monotherapy	27 (40%)
Anti-CTLA-4 + Anti-PD-1 combined	10 (15%)
Anti-PD-1 +/− Anti-CTLA-4	11 (16%)
**Immune-induced granulomatosis**	
Median time since initiation of ICI (min–max) (months)	3 (1–43)
Thoracic involvement	61 (91%)
Grade 1 impairment	38 (63%)
Grade 2 impairment	17 (28%)
Grade 3 or 4 impairment	6 (10%)
Dermatological involvement	32 (48%)
Lymph node invasion	8 (12%)
Hepatic involvement	2 (3%)
Bone involvement	5 (7%)
Histological confirmation	62 (93%)
Therapeutic management	
Systemic corticosteroids	25 (37%)
Discontinuation of immunotherapy	33 (49%)
Granulomatosis outcome	
Radiological outcome of granulomatosis	N = 62
Stability	8 (13%)
Partial or complete regression	54 (87%)
**Oncological outcome at the time of granulomatosis**	
Patient in adjuvant condition	N = 25
Relapse	3 (12%)
Patients in metastatic stage	N = 42
Objective response	29 (69%)
Complete response	16 (55%)
Partial response	13 (45%)
Stability	5 (12%)
Progression	8 (19%)
**Oncological outcome at last reported evaluation**	
Mean follow-up time for melanoma (min–max) (months)	8 (1–34)
Patient in adjuvant condition	N = 25
Relapse	3 (12%)
Patients in metastatic stage	N = 42
Objective response	24 (57%)
Complete response	16 (67%)
Partial response	8 (33%)
Stability	4 (10%)
Progression	14 (33%)

## Data Availability

The data presented in this study are available upon request from the corresponding author.

## References

[1] Schadendorf D., Hodi F.S., Robert C., Weber J.S., Margolin K., Hamid O., Patt D., Chen T.-T., Berman D.M., Wolchok J.D. (2015). Pooled Analysis of Long-Term Survival Data from Phase II and Phase III Trials of Ipilimumab in Unresectable or Metastatic Melanoma. J. Clin. Oncol..

[2] Robert C., Long G.V., Brady B., Dutriaux C., Maio M., Mortier L., Hassel J.C., Rutkowski P., McNeil C., Kalinka-Warzocha E. (2015). Nivolumab in Previously Untreated Melanoma without BRAF Mutation. N. Engl. J. Med..

[3] Larkin J., Chiarion-Sileni V., Gonzalez R., Grob J.-J., Rutkowski P., Lao C.D., Cowey C.L., Schadendorf D., Wagstaff J., Dummer R. (2019). Five-Year Survival with Combined Nivolumab and Ipilimumab in Advanced Melanoma. N. Engl. J. Med..

[4] National Cancer Institute (2006). Common Terminology Criteria for Adverse Events v3.0 (CTCAE).

[5] Cohen P.R., Kurzrock R. (2007). Sarcoidosis and Malignancy. Clin. Dermatol..

[6] Bonifazi M., Bravi F., Gasparini S., La Vecchia C., Gabrielli A., Wells A.U., Renzoni E.A. (2015). Sarcoidosis and Cancer Risk: Systematic Review and Meta-Analysis of Observational Studies. Chest.

[7] Askling J., Grunewald J., Eklund A., Hillerdal G., Ekbom A. (1999). Increased Risk for Cancer Following Sarcoidosis. Am. J. Respir. Crit. Care Med..

[8] Seve P., Schott A.M., Pavic M., Broussolle C., Gilis L., Thomas L. (2009). Sarcoidosis and Melanoma: A Referral Center Study of 1,199 Cases. Dermatology.

[9] Beutler B.D., Cohen P.R. (2015). Sarcoidosis in Melanoma Patients: Case Report and Literature Review. Cancers.

[10] Robert C., Schoenlaub P., Avril M.F., Lok C., Grosshans E., Valeyre D., Bourgeois C., Pinquier L., Dubertret L., Guillaume J.C. (1997). Malignant Melanoma and Granulomatosis. Br. J. Dermatol..

[11] Echigo T., Saito A., Takehara K., Takata M., Hatta N. (2003). Coexistence of Micrometastatic Melanoma Cells and Sarcoid Granulomas in All Regional Lymph Nodes in a Patient with Acral Melanoma. Clin. Exp. Dermatol..

[12] Rubinstein I., Baum G.L., Yellin A., Herczeg E. (1985). Sarcoidosis: A Cause of Bilateral Hilar Lymphadenopathy after Excision of Malignant Melanoma of the Arm. South. Med. J..

[13] Sacks E.L., Donaldson S.S., Gordon J., Dorfman R.F. (1978). Epithelioid Granulomas Associated with Hodgkin’s Disease: Clinical Correlations in 55 Previously Untreated Patients. Cancer.

[14] Miedema J., Nunes H. (2021). Drug-Induced Sarcoidosis-like Reactions. Curr. Opin. Pulm. Med..

[15] Cleven K.L., Ye K., Zeig-Owens R., Hena K.M., Montagna C., Shan J., Hosgood H.D., Jaber N., Weiden M.D., Colbeth H.L. (2019). Genetic Variants Associated with FDNY WTC-Related Sarcoidosis. Int. J. Environ. Res. Public Health.

[16] Kishore A., Petrek M. (2018). Next-Generation Sequencing Based HLA Typing: Deciphering Immunogenetic Aspects of Sarcoidosis. Front. Genet..

[17] Grunewald J., Spagnolo P., Wahlström J., Eklund A. (2015). Immunogenetics of Disease-Causing Inflammation in Sarcoidosis. Clin. Rev. Allergy Immunol..

[18] Koth L.L., Solberg O.D., Peng J.C., Bhakta N.R., Nguyen C.P., Woodruff P.G. (2011). Sarcoidosis Blood Transcriptome Reflects Lung Inflammation and Overlaps with Tuberculosis. Am. J. Respir. Crit. Care Med..

[19] Gupta D., Agarwal R., Aggarwal A.N., Jindal S.K. (2007). Molecular Evidence for the Role of Mycobacteria in Sarcoidosis: A Meta-Analysis. Eur. Respir. J..

[20] Song Z., Marzilli L., Greenlee B.M., Chen E.S., Silver R.F., Askin F.B., Teirstein A.S., Zhang Y., Cotter R.J., Moller D.R. (2005). Mycobacterial Catalase-Peroxidase Is a Tissue Antigen and Target of the Adaptive Immune Response in Systemic Sarcoidosis. J. Exp. Med..

[21] Milman N., Lisby G., Friis S., Kemp L. (2004). Prolonged Culture for Mycobacteria in Mediastinal Lymph Nodes from Patients with Pulmonary Sarcoidosis. A Negative Study. Sarcoidosis Vasc. Diffus. Lung Dis..

[22] Eishi Y., Suga M., Ishige I., Kobayashi D., Yamada T., Takemura T., Takizawa T., Koike M., Kudoh S., Costabel U. (2002). Quantitative Analysis of Mycobacterial and Propionibacterial DNA in Lymph Nodes of Japanese and European Patients with Sarcoidosis. J. Clin. Microbiol..

[23] Ebe Y., Ikushima S., Yamaguchi T., Kohno K., Azuma A., Sato K., Ishige I., Usui Y., Takemura T., Eishi Y. (2000). Proliferative Response of Peripheral Blood Mononuclear Cells and Levels of Antibody to Recombinant Protein from Propionibacterium Acnes DNA Expression Library in Japanese Patients with Sarcoidosis. Sarcoidosis Vasc. Diffus. Lung Dis..

[24] Song J., Zhao M., Li Q., Lu L., Zhou Y., Zhang Y., Chen T., Tang D., Zhou N., Yin C. (2019). IL-17A Can Promote Propionibacterium acnes-Induced Sarcoidosis-Like Granulomatosis in Mice. Front. Immunol..

[25] Ishige I., Eishi Y., Takemura T., Kobayashi I., Nakata K., Tanaka I., Nagaoka S., Iwai K., Watanabe K., Takizawa T. (2005). Propionibacterium Acnes Is the Most Common Bacterium Commensal in Peripheral Lung Tissue and Mediastinal Lymph Nodes from Subjects without Sarcoidosis. Sarcoidosis Vasc. Diffus. Lung Dis..

[26] Kreider M.E., Christie J.D., Thompson B., Newman L., Rose C., Barnard J., Bresnitz E., Judson M.A., Lackland D.T., Rossman M.D. (2005). Relationship of Environmental Exposures to the Clinical Phenotype of Sarcoidosis. Chest.

[27] Newman L.S., Rose C.S., Bresnitz E.A., Rossman M.D., Barnard J., Frederick M., Terrin M.L., Weinberger S.E., Moller D.R., McLennan G. (2004). A Case Control Etiologic Study of Sarcoidosis: Environmental and Occupational Risk Factors. Am. J. Respir. Crit. Care Med..

[28] Eckert A., Schoeffler A., Dalle S., Phan A., Kiakouama L., Thomas L. (2009). Anti-CTLA4 Monoclonal Antibody Induced Sarcoidosis in a Metastatic Melanoma Patient. Dermatology.

[29] Berthod G., Lazor R., Letovanec I., Romano E., Noirez L., Mazza Stalder J., Speiser D.E., Peters S., Michielin O. (2012). Pulmonary Sarcoid-Like Granulomatosis Induced by Ipilimumab. J. Clin. Oncol..

[30] Vogel W.V., Guislain A., Kvistborg P., Schumacher T.N.M., Haanen J.B.A.G., Blank C.U. (2012). Ipilimumab-Induced Sarcoidosis in a Patient with Metastatic Melanoma Undergoing Complete Remission. J. Clin. Oncol..

[31] Wilgenhof S., Morlion V., Seghers A.C., Du Four S., Vanderlinden E., Hanon S., Vandenbroucke F., Everaert H., Neyns B. (2012). Sarcoidosis in a Patient with Metastatic Melanoma Sequentially Treated with Anti-CTLA-4 Monoclonal Antibody and Selective BRAF Inhibitor. Anticancer Res..

[32] Reule R.B., North J.P. (2013). Cutaneous and Pulmonary Sarcoidosis-like Reaction Associated with Ipilimumab. J. Am. Acad. Dermatol..

[33] Andersen R., Nørgaard P., Al-Jailawi M.K.M., Svane I.M. (2014). Late Development of Splenic Sarcoidosis-like Lesions in a Patient with Metastatic Melanoma and Long-Lasting Clinical Response to Ipilimumab. Oncoimmunology.

[34] Murphy K.P., Kennedy M.P., Barry J.E., O’Regan K.N., Power D.G. (2014). New-Onset Mediastinal and Central Nervous System Sarcoidosis in a Patient with Metastatic Melanoma Undergoing CTLA4 Monoclonal Antibody Treatment. Oncol. Res. Treat..

[35] Danlos F.-X., Pagès C., Baroudjian B., Vercellino L., Battistella M., Mimoun M., Jebali M., Bagot M., Tazi A., Lebbé C. (2016). Nivolumab-Induced Sarcoid-Like Granulomatous Reaction in a Patient with Advanced Melanoma. Chest.

[36] Martínez Leboráns L., Esteve Martínez A., Victoria Martínez A.M., Alegre de Miquel V., Berrocal Jaime A. (2016). Cutaneous Sarcoidosis in a Melanoma Patient under Ipilimumab Therapy. Dermatol. Ther..

[37] Koelzer V.H., Rothschild S.I., Zihler D., Wicki A., Willi B., Willi N., Voegeli M., Cathomas G., Zippelius A., Mertz K.D. (2016). Systemic Inflammation in a Melanoma Patient Treated with Immune Checkpoint Inhibitors-an Autopsy Study. J. Immunother. Cancer.

[38] Reuss J.E., Kunk P.R., Stowman A.M., Gru A.A., Slingluff C.L., Gaughan E.M. (2016). Sarcoidosis in the Setting of Combination Ipilimumab and Nivolumab Immunotherapy: A Case Report & Review of the Literature. J. Immunother. Cancer.

[39] Montaudié H., Pradelli J., Passeron T., Lacour J.-P., Leroy S. (2017). Pulmonary Sarcoid-like Granulomatosis Induced by Nivolumab. Br. J. Dermatol..

[40] Reddy S.B., Possick J.D., Kluger H.M., Galan A., Han D. (2017). Sarcoidosis Following Anti-PD-1 and Anti-CTLA-4 Therapy for Metastatic Melanoma. J. Immunother..

[41] Dunn-Pirio A.M., Shah S., Eckstein C. (2018). Neurosarcoidosis Following Immune Checkpoint Inhibition. Case Rep. Oncol..

[42] Nishino M., Sholl L.M., Awad M.M., Hatabu H., Armand P., Hodi F.S. (2018). Sarcoid-Like Granulomatosis of the Lung Related to Immune-Checkpoint Inhibitors: Distinct Clinical and Imaging Features of a Unique Immune-Related Adverse Event. Cancer Immunol. Res..

[43] Faviez G., Bousquet E., Rabeau A., Rouquette I., Collot S., Goumarre C., Meyer N., Prevot G., Mazieres J. (2018). Sarcoid-like granulomatosis in cancer patients treated with immune checkpoints inhibitors. Rev. Mal. Respir..

[44] Laroche A., Alarcon Chinchilla E., Bourgeault E., Doré M.-A. (2018). Erythema Nodosum as the Initial Presentation of Nivolumab-Induced Sarcoidosis-Like Reaction. J. Cutan. Med. Surg..

[45] Yatim N., Mateus C., Charles P. (2018). Sarcoidosis Post-Anti-PD-1 Therapy, Mimicking Relapse of Metastatic Melanoma in a Patient Undergoing Complete Remission. Rev. Med. Interne.

[46] Jespersen H., Bjursten S., Ny L., Levin M. (2018). Checkpoint Inhibitor-Induced Sarcoid Reaction Mimicking Bone Metastases. Lancet Oncol..

[47] Dimitriou F., Frauchiger A.L., Urosevic-Maiwald M., Naegeli M.C., Goldinger S.M., Barysch M., Franzen D., Kamarachev J., Braun R., Dummer R. (2018). Sarcoid-like Reactions in Patients Receiving Modern Melanoma Treatment. Melanoma Res..

[48] Lu Y. (2019). FDG PET/CT Course of Pembrolizumab-Associated Multiorgan Sarcoidosis. Clin. Nucl. Med..

[49] Tetzlaff M.T., Nelson K.C., Diab A., Staerkel G.A., Nagarajan P., Torres-Cabala C.A., Chasen B.A., Wargo J.A., Prieto V.G., Amaria R.N. (2018). Granulomatous/Sarcoid-like Lesions Associated with Checkpoint Inhibitors: A Marker of Therapy Response in a Subset of Melanoma Patients. J. Immunother. Cancer.

[50] Fukuchi K., Hikawa M., Sano Y., Kasuya A., Aoshima M., Tatsuno K., Nakamura Y., Kosugi I., Tokura Y. (2019). Sarcoid-like Reaction and Vitiligo Occurring after Nivolumab Therapy in a Patient with Metastatic Melanoma. J. Dermatol..

[51] Cervantes J., Rosen A., Dehesa L., Dickinson G., Alonso-Llamazares J. (2019). Granulomatous Reaction in a Patient with Metastatic Melanoma Treated with Ipilimumab: First Case Reported with Isolated Cutaneous Findings. Actas Dermo-Sifiliográficas.

[52] Toumeh A., Sakhi R., Shah S., Arudra S.K.C., De Las Casas L.E., Skeel R.T. (2016). Ipilimumab-Induced Granulomatous Disease Occurring Simultaneously with Disease Progression in a Patient with Metastatic Melanoma. Am. J. Ther..

[53] Lidar M., Giat E., Garelick D., Horowitz Y., Amital H., Steinberg-Silman Y., Schachter J., Shapira-Frommer R., Markel G. (2018). Rheumatic Manifestations among Cancer Patients Treated with Immune Checkpoint Inhibitors. Autoimmun. Rev..

[54] Burillo-Martinez S., Morales-Raya C., Prieto-Barrios M., Rodriguez-Peralto J.-L., Ortiz-Romero P.-L. (2017). Pembrolizumab-Induced Extensive Panniculitis and Nevus Regression: Two Novel Cutaneous Manifestations of the Post-Immunotherapy Granulomatous Reactions Spectrum. JAMA Dermatol..

[55] Nandavaram S., Nadkarni A. (2018). Ipilimumab-Induced Sarcoidosis and Thyroiditis. Am. J. Ther..

[56] Tan I., Malinzak M., Salama A.K.S. (2018). Delayed Onset of Neurosarcoidosis after Concurrent Ipilimumab/Nivolumab Therapy. J. Immunother. Cancer.

[57] Van Willigen W.W., Gerritsen W.R., Aarntzen E.H.J.G. (2019). 18F-FDG PET/CT of Multiorgan Sarcoid-Like Reaction During Anti-PD-1 Treatment for Melanoma. Clin. Nucl. Med..

[58] Wang L.L., Patel G., Chiesa-Fuxench Z.C., McGettigan S., Schuchter L., Mitchell T.C., Ming M.E., Chu E.Y. (2018). Timing of Onset of Adverse Cutaneous Reactions Associated with Programmed Cell Death Protein 1 Inhibitor Therapy. JAMA Dermatol..

[59] Woodbeck R., Metelitsa A.I., Naert K.A. (2018). Granulomatous Tumoral Melanosis Associated with Pembrolizumab Therapy: A Mimicker of Disease Progression in Metastatic Melanoma. Am. J. Dermatopathol..

[60] Tissot C., Carsin A., Freymond N., Pacheco Y., Devouassoux G. (2013). Sarcoidosis Complicating Anti-Cytotoxic T-Lymphocyte-Associated Antigen-4 Monoclonal Antibody Biotherapy. Eur. Respir. J..

[61] Firwana B., Ravilla R., Raval M., Hutchins L., Mahmoud F. (2017). Sarcoidosis-like Syndrome and Lymphadenopathy Due to Checkpoint Inhibitors. J. Oncol. Pharm. Pract..

[62] Rodriguez E.F., Lipson E., Suresh K., Cappelli L.C., Monaco S.E., Maleki Z. (2019). Immune Checkpoint Blocker-Related Sarcoid-like Granulomatous Inflammation: A Rare Adverse Event Detected in Lymph Node Aspiration Cytology of Patients Treated for Advanced Malignant Melanoma. Hum. Pathol..

[63] Chorti E., Kanaki T., Zimmer L., Hadaschik E., Ugurel S., Gratsias E., Roesch A., Bonella F., Wessendorf T.E., Wälscher J. (2020). Drug-Induced Sarcoidosis-like Reaction in Adjuvant Immunotherapy: Increased Rate and Mimicker of Metastasis. Eur. J. Cancer.

[64] Frohlich M., Wang H., Sakr L. (2020). Sarcoid-like Reaction Discovered on EBUS-TBNA of Intrathoracic Lymph Nodes During Immunotherapy for Metastatic Melanoma. J. Immunother..

[65] Tulbah R.I., Rowe S.P., Solnes L.B., Javadi M.S. (2019). Nivolumab-Associated Pulmonary and Bone Sarcoidosis in a Patient With Melanoma of Unknown Primary. Clin. Nucl. Med..

[66] Urrego-Callejas T., Sandoval-Álvarez S., Gómez-Wolff R., Vásquez G. (2019). Cutaneous and Pulmonary Sarcoid-Like Reaction Induced by Nivolumab: Case Report and Brief Literature Review. J. Clin. Rheumatol..

[67] Marcoval J., Bauer-Alonso A., Fornons-Servent R., Jiménez-Colomo L., Sabaté-Llobera A., Penín R.M. (2021). Subcutaneous Sarcoidosis Induced by Pembrolizumab in a Melanoma Patient Mimicking Subcutaneous Metastasis at 18F-FDG PET/CT. Rev. Esp. Med. Nucl. Imagen. Mol..

[68] Apalla Z., Kemanetzi C., Papageorgiou C., Bobos M., Manoli M., Fotiadou C., Hatzibougias D., Boukovinas I., Stergiou E., Levva S. (2021). Challenges in Sarcoidosis and Sarcoid-like Reactions Associated to Immune Checkpoint Inhibitors: A Narrative Review apropos of a Case. Dermatol. Ther..

[69] Keukeleire S.D., Schwarze J., Awada G., Everaert H., Van Binst A.M., Cras L., Neyns B., Aspeslagh S. (2020). An Atypical Sarcoid-like Reaction during Anti-Protein Death 1 Treatment in a Patient with Metastatic Melanoma. Melanoma Res..

[70] Garanzini E.M., Scaramuzza D., Spadarella G., Di Guardo L., Marchianò A. (2020). Sarcoidosis-like Disease Mimicking Metastases during Adjuvant Ipilimumab Therapy in Advanced Melanoma Patient: CT Scan and MRI Help in Managing Difficult Clinical Decision. BJR Case Rep..

[71] Mobini N., Dhillon R., Dickey J., Spoon J., Sadrolashrafi K. (2019). Exclusive Cutaneous and Subcutaneous Sarcoidal Granulomatous Inflammation Due to Immune Checkpoint Inhibitors: Report of Two Cases with Unusual Manifestations and Review of the Literature. Case Rep. Dermatol. Med..

[72] Chahin M., Stack A., Siddiqi A., Shaikh M. (2020). Is It Metastatic Melanoma or Is It Sarcoidosis? Non-Caseating Granulomas Due to Pembrolizumab. BMJ Case Rep..

[73] Abdel-Wahab N., Shah M., Suarez-Almazor M.E. (2016). Adverse Events Associated with Immune Checkpoint Blockade in Patients with Cancer: A Systematic Review of Case Reports. PLoS ONE.

[74] Bronstein Y., Ng C.S., Hwu P., Hwu W.-J. (2011). Radiologic Manifestations of Immune-Related Adverse Events in Patients with Metastatic Melanoma Undergoing Anti-CTLA-4 Antibody Therapy. Am. J. Roentgenol..

[75] Tirumani S.H., Ramaiya N.H., Keraliya A., Bailey N.D., Ott P.A., Hodi F.S., Nishino M. (2015). Radiographic Profiling of Immune-Related Adverse Events in Advanced Melanoma Patients Treated with Ipilimumab. Cancer Immunol. Res..

[76] Hodi F.S., Ribas A., Daud A., Hamid O., Robert C., Kefford R., Hwu W.-J., Gangadhar T.C., Joshua A.M., Hersey P. (2014). Patterns of Response in Patients with Advanced Melanoma Treated with Pembrolizumab (MK-3475) and Evaluation of Immune-Related Response Criteria (IrRC). J. ImmunoTherapy Cancer.

[77] Markovic S.N., Galli F., Suman V.J., Nevala W.K., Paulsen A.M., Hung J.C., Gansen D.N., Erickson L.A., Marchetti P., Wiseman G.A. (2018). Non-Invasive Visualization of Tumor Infiltrating Lymphocytes in Patients with Metastatic Melanoma Undergoing Immune Checkpoint Inhibitor Therapy: A Pilot Study. Oncotarget.

[78] Statement on Sarcoidosis (1999). Joint Statement of the American Thoracic Society (ATS), the European Respiratory Society (ERS) and the World Association of Sarcoidosis and Other Granulomatous Disorders (WASOG) Adopted by the ATS Board of Directors and by the ERS Executive Committee, February 1999. Am. J. Respir. Crit. Care Med..

[79] Judson M.A. (2014). Advances in the Diagnosis and Treatment of Sarcoidosis. F1000Prime Rep.

[80] Hao W. (2014). Mathematical Model of Sarcoidosis. Proc. Natl. Acad. Sci. USA.

[81] Fischer A., Ellinghaus D., Nutsua M., Hofmann S., Montgomery C.G., Iannuzzi M.C., Rybicki B.A., Petrek M., Mrazek F., Pabst S. (2015). Identification of Immune-Relevant Factors Conferring Sarcoidosis Genetic Risk. Am. J. Respir. Crit. Care Med..

[82] Cinetto F., Agostini C. (2016). Advances in Understanding the Immunopathology of Sarcoidosis and Implications on Therapy. Expert Rev. Clin. Immunol..

[83] Facco M., Cabrelle A., Teramo A., Olivieri V., Gnoato M., Teolato S., Ave E., Gattazzo C., Fadini G.P., Calabrese F. (2011). Sarcoidosis Is a Th1/Th17 Multisystem Disorder. Thorax.

[84] Facco M., Baesso I., Miorin M., Bortoli M., Cabrelle A., Boscaro E., Gurrieri C., Trentin L., Zambello R., Calabrese F. (2007). Expression and Role of CCR6/CCL20 Chemokine Axis in Pulmonary Sarcoidosis. J. Leukoc. Biol..

[85] Li Q., Laumonnier Y., Syrovets T., Simmet T. (2013). Recruitment of CCR6-Expressing Th17 Cells by CCL20 Secreted from Plasmin-Stimulated Macrophages. Acta Biochim. Biophys. Sin..

[86] Ramstein J., Broos C.E., Simpson L.J., Ansel K.M., Sun S.A., Ho M.E., Woodruff P.G., Bhakta N.R., Christian L., Nguyen C.P. (2016). IFN-γ-Producing T-Helper 17.1 Cells Are Increased in Sarcoidosis and Are More Prevalent than T-Helper Type 1 Cells. Am. J. Respir. Crit. Care Med..

[87] Huang H., Lu Z., Jiang C., Liu J., Wang Y., Xu Z. (2013). Imbalance between Th17 and Regulatory T-Cells in Sarcoidosis. Int. J. Mol. Sci..

[88] Mortaz E., Rezayat F., Amani D., Kiani A., Garssen J., Adcock I.M., Velayati A. (2016). The Roles of T Helper 1, T Helper 17 and Regulatory T Cells in the Pathogenesis of Sarcoidosis. Iran. J. Allergy Asthma Immunol..

[89] Moller D.R., Rybicki B.A., Hamzeh N.Y., Montgomery C.G., Chen E.S., Drake W., Fontenot A.P. (2017). Genetic, Immunologic, and Environmental Basis of Sarcoidosis. Ann. Am. Thorac. Soc..

[90] Gkiozos I., Kopitopoulou A., Kalkanis A., Vamvakaris I.N., Judson M.A., Syrigos K.N. (2018). Sarcoidosis-Like Reactions Induced by Checkpoint Inhibitors. J. Thorac. Oncol..

[91] Ying H., Yang L., Qiao G., Li Z., Zhang L., Yin F., Xie D., Zhang J. (2010). Cutting Edge: CTLA-4–B7 Interaction Suppresses Th17 Cell Differentiation. J. Immunol..

[92] Von Euw E., Chodon T., Attar N., Jalil J., Koya R.C., Comin-Anduix B., Ribas A. (2009). CTLA4 Blockade Increases Th17 Cells in Patients with Metastatic Melanoma. J. Transl. Med..

[93] Broos C.E., van Nimwegen M., In ’t Veen J.C.C.M., Hoogsteden H.C., Hendriks R.W., van den Blink B., Kool M. (2015). Decreased Cytotoxic T-Lymphocyte Antigen 4 Expression on Regulatory T Cells and Th17 Cells in Sarcoidosis: Double Trouble?. Am. J. Respir. Crit. Care Med..

[94] Parry R.V., Chemnitz J.M., Frauwirth K.A., Lanfranco A.R., Braunstein I., Kobayashi S.V., Linsley P.S., Thompson C.B., Riley J.L. (2005). CTLA-4 and PD-1 Receptors Inhibit T-Cell Activation by Distinct Mechanisms. Mol. Cell. Biol..

[95] D’Addio F., Riella L.V., Mfarrej B.G., Chabtini L., Adams L.T., Yeung M., Yagita H., Azuma M., Sayegh M.H., Guleria I. (2011). The Link between the PDL1 Costimulatory Pathway and Th17 in Fetomaternal Tolerance. J. Immunol..

[96] Zhang Y., Liu Z., Tian M., Hu X., Wang L., Ji J., Liao A. (2018). The Altered PD-1/PD-L1 Pathway Delivers the ‘One-Two Punch’ Effects to Promote the Treg/Th17 Imbalance in Pre-Eclampsia. Cell. Mol. Immunol..

[97] Georas S.N., Chapman T.J., Crouser E.D. (2016). Sarcoidosis and T-Helper Cells. Th1, Th17, or Th17.1?. Am. J. Respir. Crit. Care Med..

[98] Ten Berge B., Paats M.S., Bergen I.M., van den Blink B., Hoogsteden H.C., Lambrecht B.N., Hendriks R.W., Kleinjan A. (2012). Increased IL-17A Expression in Granulomas and in Circulating Memory T Cells in Sarcoidosis. Rheumatology.

[99] Lomax A.J., McGuire H.M., McNeil C., Choi C.J., Hersey P., Karikios D., Shannon K., van Hal S., Carr U., Crotty A. (2017). Immunotherapy-Induced Sarcoidosis in Patients with Melanoma Treated with PD-1 Checkpoint Inhibitors: Case Series and Immunophenotypic Analysis. Int. J. Rheum. Dis..

[100] Linke M., Pham H.T.T., Katholnig K., Schnöller T., Miller A., Demel F., Schütz B., Rosner M., Kovacic B., Sukhbaatar N. (2017). Chronic Signaling via the Metabolic Checkpoint Kinase MTORC1 Induces Macrophage Granuloma Formation and Marks Sarcoidosis Progression. Nat. Immunol..

